# Effect of canagliflozin on N-terminal pro-brain natriuretic peptide in patients with type 2 diabetes and chronic heart failure according to baseline use of glucose-lowering agents

**DOI:** 10.1186/s12933-021-01369-5

**Published:** 2021-09-03

**Authors:** Atsushi Tanaka, Shigeru Toyoda, Takumi Imai, Kazuki Shiina, Hirofumi Tomiyama, Yasushi Matsuzawa, Takahiro Okumura, Yumiko Kanzaki, Katsuya Onishi, Arihiro Kiyosue, Masami Nishino, Yasushi Sakata, Koichi Node, the CANDLE trial investigators

**Affiliations:** 1 Department of Cardiovascular Medicine, Saga University, 5-1-1 Nabeshima, Saga, 849-8501 Japan; 2 Department of Cardiovascular Medicine, Dokkyo Medical University School of Medicine, Mibu, Japan; 3 Department of Medical Statistics, Graduate School of Medicine, Osaka City University, Osaka, Japan; 4 Department of Cardiology, Tokyo Medical University, Tokyo, Japan; 5 Division of Cardiology, Yokohama City University Medical Center, Yokohama, Japan; 6 Department of Cardiology, Nagoya University Graduate School of Medicine, Nagoya, Japan; 7 Department of Cardiology, Osaka Medical and Pharmaceutical University, Takatsuki, Japan; 8 Onishi Heart Clinic, Tsu, Japan; 9 Department of Cardiology, Moriyama Memorial Hospital, Tokyo, Japan; 10 Division of Cardiology, Osaka Rosai Hospital, Sakai, Japan; 11 Department of Cardiovascular Medicine, Osaka University Graduate School of Medicine, Osaka, Japan

**Keywords:** Type 2 diabetes, Chronic heart failure, Sodium–glucose cotransporter 2 inhibitor, Metformin, Dipeptidyl peptidase-4 inhibitor

## Abstract

**Background:**

Sodium–glucose cotransporter 2 (SGLT2) inhibitors reduce the risk of a deterioration in heart failure (HF) and mortality in patients with a broad range of cardiovascular risks. Recent guidelines recommend considering the use of SGLT2 inhibitors in patients with type 2 diabetes (T2D) and HF, irrespective of their glycemic control status and background use of other glucose-lowering agents including metformin. However, only a small number of studies have investigated whether the effects of SGLT2 inhibitor in these patients differ by the concomitant use of other glucose-lowering agents.

**Methods:**

This was a post-hoc analysis of the CANDLE trial (UMIN000017669), an investigator-initiated, multicenter, open-label, randomized, controlled trial. The primary aim of the analysis was to assess the effect of 24 weeks of treatment with canagliflozin, relative to glimepiride, on N-terminal pro-brain natriuretic peptide (NT-proBNP) concentration in patients with T2D and clinically stable chronic HF. In the present analysis, the effect of canagliflozin on NT-proBNP concentration was assessed in the patients according to their baseline use of other glucose-lowering agents.

**Results:**

Almost all patients in the CANDLE trial presented as clinically stable (New York Heart Association class I to II), with about 70% of participants having HF with a preserved ejection fraction phenotype (defined as a left ventricular ejection fraction ≥ 50%) at baseline. Of the 233 patients randomized to either canagliflozin (100 mg daily) or glimepiride (starting dose 0.5 mg daily), 85 (36.5%) had not been taking any glucose-lowering agents at baseline (naïve). Of the 148 patients who had been taking at least one glucose-lowering agent at baseline (non-naïve), 44 (29.7%) and 127 (85.8%) had received metformin or a dipeptidyl dipeptidase-4 (DPP-4) inhibitor, respectively. The group ratio (canagliflozin vs. glimepiride) of proportional changes in the geometric means of NT-proBNP concentration was 0.95 (95% confidence interval [CI] 0.76 to 1.18, *p* = 0.618) for the naïve subgroup, 0.92 (95% CI 0.79 to1.07, *p* = 0.288) for the non-naïve subgroup, 0.90 (95% CI 0.68 to 1.20, *p* = 0.473) for the metformin-user subgroup, and 0.91 (95% CI 0.77 to 1.08, *p* = 0.271) for the DPP-4 inhibitor-user subgroup. No heterogeneity in the effect of canagliflozin, relative to glimepiride, on NT-proBNP concentration was observed in the non-naïve subgroups compared to that in the naïve subgroup.

**Conclusion:**

The impact of canagliflozin treatment on NT-proBNP concentration appears to be independent of the background use of diabetes therapy in the patient population examined.

*Trial registration* University Medical Information Network Clinical Trial Registry, number 000017669. Registered on May 25, 2015

**Supplementary Information:**

The online version contains supplementary material available at 10.1186/s12933-021-01369-5.

## Introduction

Metformin has been used widely for a long period of time and is an established and fundamental glucose-lowering agent for the treatment of type 2 diabetes (T2D) [1–3]. Several observational studies have demonstrated the clinical benefits and safety of metformin therapy even in patients with T2D complicated by heart failure (HF) [4–7]. Earlier cardiovascular outcome trials (CVOT) on sodium–glucose cotransporter 2 (SGLT2) inhibitors showed they markedly reduced the risk of hospitalization for HF (HHF) and mortality in patients with T2D and established atherosclerotic cardiovascular diseases (ASCVD) or at high-risk of cardiovascular events [8–10]. These striking findings led the European Society of Cardiology (ESC) in collaboration with the European Association for the Study of Diabetes (EASD) to develop guidelines for diabetes, pre-diabetes, and cardiovascular diseases. The guidelines recommended the use of SGLT2 inhibitors in these high-risk patient populations, regardless of prior use of metformin [11]. Although the proportion of patients with concomitant HF at baseline in these studies was small, a significant benefit was observed in subsequent CVOTs specifically in HF patients with a reduced ejection fraction (HFrEF) [12]. As a consequence, the latest American Diabetes Association (ADA) guidelines recommend the use of SGLT2 inhibitors in patients with T2D and HF, especially HFrEF, regardless of the patient’s diabetes status and prior use of metformin [13]. This will result in SGLT2 inhibitors being used more frequently for HF care and in patients with a variety of clinical backgrounds and use of medications.

The majority of patients with T2D recruited in these earlier CVOTs on SGLT2 inhibitors were treated with metformin at baseline. Nevertheless, treatment with SGLT2 inhibitors consistently reduced the risk of adverse cardiovascular events, such as HHF and cardiovascular death, in patients who had received a variety of glucose-lowering agents at baseline, including metformin [14–16]. However, the proportion of participants with concomitant HF was small in these CVOTs, and there is limited evidence as to whether SGLT2 inhibitors have different effects on HF-related parameters in patients with T2D and concomitant HF, according to their use of glucose-lowering agents.

The CANDLE trial in clinically stable patients with T2D and documented chronic HF (CHF) was designed primarily to assess the clinical safety and efficacy of 24 weeks of add-on canagliflozin treatment, relative to glimepiride, based on the effects on N-terminal pro-brain natriuretic peptide (NT-proBNP) concentration [17, 18]. This paper reports the findings of a post hoc analysis of the CANDLE trial that examined whether the effect of canagliflozin on HF-related markers, including NT-proBNP, was affected by the baseline status of T2D medications.

## Methods

### Study design and participants

This was a post hoc analysis of the CANDLE trial (UMIN000017669), an investigator-initiated, multicenter, prospective, randomized, open-label trial undertaken at 34 centers in Japan [18]. The details of the design and inclusion/exclusion criteria have been reported elsewhere [17, 18]. In brief, adults with T2D and CHF categorized as New York Heart Association (NYHA) class I to III and clinically stable without changes in NYHA class or CHF medications four weeks prior to eligibility assessment, were assigned randomly to either canagliflozin (100 mg daily) or glimepiride (starting dose 0.5 mg daily) groups. Randomization was carried out using a web-based allocation system and the minimization method balanced for age (< 65, ≥ 65 yr), HbA1c level (< 6.5%, ≥ 6.5%), and left ventricular ejection fraction (LVEF; < 40%, ≥ 40%) at the time of screening. Key exclusion criteria were severe renal impairment (estimated glomerular filtration rate < 45 mL/min/1.73m^2^ or on dialysis), NYHA class IV, low body mass index (BMI; < 18.5 kg/m^2^), and a recent history of coronary artery disease needing revascularization or a stroke within 3 months prior to screening.

The trial was approved by the institutional review boards of the individual sites and conducted in accordance with the Declaration of Helsinki. All participants provided written, informed consent prior to screening and randomization.

### Measurements and endpoints

The details of the original outcome measures in the CANDLE trial have been described previously [17, 18]. The post hoc analysis compared the -inter or -intra group ratios or differences in changes from baseline to week 24 in NT-proBNP concentration (primary endpoint in the present study), office systolic blood pressure (SBP), BMI, estimated plasma volume (ePV), and NYHA class, with the data stratified according to the use of glucose-lowering agents at baseline. Patients who had not been taking any glucose-lowering agents prior to randomization were categorized to a subgroup (naïve), while those who had been taking at least one glucose-lowering agent prior to randomization were categorized to another subgroup (non-naïve). NT-proBNP concentrations were assessed at each local site and measured in a blinded manner at a central core laboratory (SRL, Inc. Tokyo, Japan) using an electrochemiluminescence immunoassay (Roche, Basel, Switzerland). The percentage change in ePV from baseline to week 24 was calculated using the Strauss formula [19, 20] as:

$${100}\times \frac{\text{hemoglobin }(\text{at baseline})}{\text{hemoglobin }(\text{at week 24})}\times \frac{1-\text{hematocrit }(\text{at week 24})}{1-\text{hematocrit }(\text{at baseline})}-{100}$$

### Statistical analysis

The efficacy analyses were conducted on the full analysis set, which included all participants who had received at least one dose of the study treatment after randomization and had no serious violation of the protocol. The baseline demographics and clinical characteristics were expressed as numbers (percentages) for categorical variables and as means ± standard deviation for continuous variables. Participants who had data at both baseline and week 24 were included in the analyses of changes in each variable. Data on NT-proBNP concentration were expressed as geometric mean (95% confidence interval [CI]), and the proportional changes from baseline to week 24 estimated using a natural logarithmic scale. Comparisons between the treatment groups were made using linear regression models for continuous outcomes and the Wilcoxon rank-sum test for changes in NYHA classification. All statistical analyses were carried out using R software, version 3.6.3 (R Foundation for Statistical Computing) at a two-sided significance level of 0.05. No adjustment for multiplicity was considered in the post hoc sub-analysis.

## Results

### Baseline demographics and clinical characteristics

**Fig. 1:**
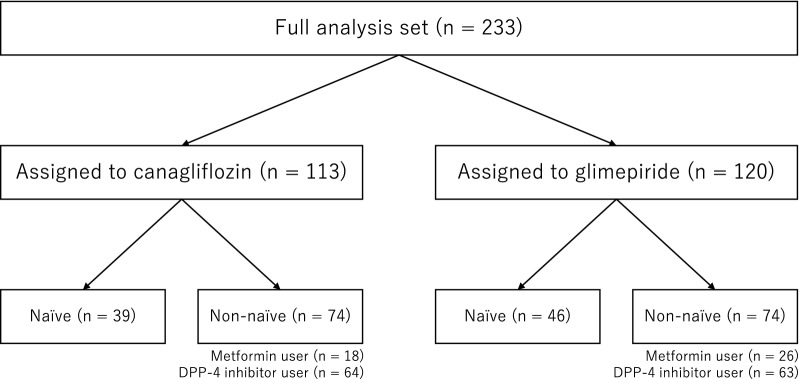
Flow-chart for the analyses carried out in the participants during the study. *DPP-4* dipeptidyl peptidase-4

A total of 245 patients in the CANDLE trial were assigned randomly to the canagliflozin (n = 122) and glimepiride (n = 123) groups, with 113 patients receiving canagliflozin and 120 receiving glimepiride included in the full analysis set [18]. The detailed baseline demographic and characteristics of the patients have been described previously [18]. Table 1 shows the baseline demographics and clinical characteristics for the full analysis set, stratified by naïve or non-naïve for baseline use of glucose-lowering agents (Fig. 1). In the CANDLE trial, almost all patients presented as clinically stable (NYHA class I to II), with about 70% of participants having HF with a preserved ejection fraction (HFpEF: defined as a LVEF ≥ 50%) phenotype at baseline. Overall, a total of 85 participants (36.5%) had not been taking any glucose-lowering agents at baseline (naïve). Of the remaining 148 patients who had been taking at least one glucose-lowering agents at baseline (non-naïve), 92 patients (canagliflozin n = 50, glimepiride n = 42) were taking only one glucose-lowering agent at baseline, while 56 patients (canagliflozin n = 24, glimepiride n = 32) were taking multiple glucose-lowering agents. In the non-naive subgroup, 44 patients (29.7%) had received metformin and 127 (85.8%) had received a dipeptidyl dipeptidase-4 (DPP-4) inhibitor (Fig. 1). The baseline demographics and clinical characteristics in each cohort (overall, naïve, and non-naïve) are also shown in Additional file 1, with almost clinical variables being relatively balanced between the naïve and non-naïve cohorts.

**Table 1:** Baseline demographic and clinical characteristics of the patients

Variables	Naïve		Non-naïve	
	Canagliflozin (n = 39)	Glimepiride (n = 46)	Canagliflozin (n = 74)	Glimepiride (n = 74)
Age, year	71.8 ± 7.6	68.9 ± 10.8	66.4 ± 10.3	68.9 ± 10.2
Males	30 (76.9)	32 (69.6)	58 (78.4)	54 (73.0)
History				
Hypertension	19 (48.7)	18 (39.1)	30 (40.5)	35 (47.3)
Dyslipidemia	19 (48.7)	21 (45.7)	27 (36.5)	33 (44.6)
Myocardial infarction	13 (33.3)	6 (13.0)	19 (25.7)	18 (24.3)
Angina pectoris	5 (12.8)	14 (30.4)	19 (25.7)	13 (17.6)
Heart failure cause				
Ischemia	16 (41.0)	17 (37.0)	38 (51.4)	29 (39.2)
Heart failure status				
NYHA class				
I	20 (51.3)	28 (60.9)	52 (70.3)	48 (64.9)
II	19 (48.7)	15 (32.6)	20 (27.0)	25 (33.8)
III	0 (0.0)	2 (4.3)	2 (2.7)	1 (1.4)
Unknown	0 (0.0)	1 (2.2)	0 (0.0)	0 (0.0)
LVEF distribution				
< 30%	2 (5.1)	2 (4.3)	3 (4.1)	6 (8.1)
30 to < 40%	5 (12.8)	3 (6.5)	6 (8.2)	6 (8.1)
40 to < 50%	5 (12.8)	6 (13.0)	13 (17.8)	10 (13.5)
≥ 50%	27 (69.2)	35 (76.1)	51 (69.9)	52 (70.3)
Medications				
Non-diabetic				
ACE inhibitor or ARB	28 (71.8)	30 (65.2)	61 (82.4)	58 (78.4)
Beta-blocker	32 (82.1)	35 (76.1)	50 (67.6)	47 (63.5)
MRA	15 (38.5)	19 (41.3)	27 (36.5)	25 (33.8)
Diuretic	15 (38.5)	25 (54.3)	31 (41.9)	28 (37.8)
Diabetic				
Insulin	0 (0.0)	0 (0.0)	4 (5.4)	3 (4.1)
Metformin	0 (0.0)	0 (0.0)	18 (24.3)	26 (35.1)
DPP-4 inhibitor	0 (0.0)	0 (0.0)	64 (86.5)	63 (85.1)
Others	0 (0.0)	0 (0.0)	16 (21.6)	25 (33.8)

Data are expressed as the mean ± standard deviation or n (%)

*ACE* angiotensin-converting enzyme, *ARB* angiotensin receptor blocker, *DPP-4* dipeptidyl peptidase-4, *LVEF* left ventricular ejection fraction, *MRA* mineralocorticoid receptor antagonist, *NYHA* New York Heart Association

### Effect on NT-proBNP concentration

**Fig. 2:**
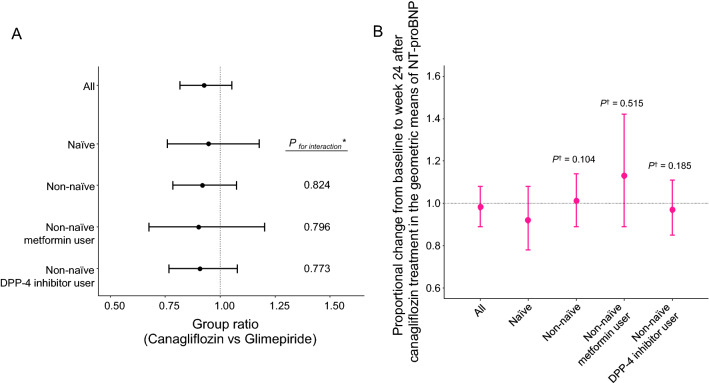
Changes in NT-proBNP concentration from baseline to week 24 in the subgroups stratified by the baseline use of glucose-lowering agents. **A** The group ratio (canagliflozin vs. glimepiride) of proportional changes from baseline to week 24 in the geometric means of NT-proBNP concentration (* refers to the naïve subgroup). **B** The proportional changes from baseline to week 24 after canagliflozin treatment in the geometric means of NT-proBNP concentration (^†^ refers to the naïve subgroup). DPP-4, dipeptidyl peptidase-4; NT-proBNP, N-terminal pro-brain natriuretic peptide

Table 2 shows the geometric means of NT-proBNP concentration at baseline and week 24 and the proportional changes from baseline to week 24, according to stratification by the baseline use of glucose-lowering agents. The group ratio (canagliflozin vs. glimepiride) of proportional changes in the geometric means of NT-proBNP concentration was 0.93 (95% CI 0.82 to 1.05, *p* = 0.244) for all patients, 0.95 (95% CI 0.76 to 1.18, *p* = 0.618) for the naïve subgroup, 0.92 (95% CI 0.79 to 1.07, *p* = 0.288) for the non-naïve subgroup, 0.90 (95% CI 0.68 to 1.20, *p* = 0.473) for the non-naïve metformin-user subgroup, and 0.91 (95% CI 0.77 to 1.08, *p* = 0.271) for the non-naïve DPP-4 inhibitor-user subgroup (Fig. 2A). No heterogeneity in the effect of canagliflozin relative to glimepiride on NT-proBNP concentration was observed in any of the three non-naïve subgroups, compared to that in the naïve subgroup (Fig. 2A). In the canagliflozin group, the proportional change from baseline to week 24 in the geometric mean of NT-proBNP concentration in the naïve subgroup was numerically smaller than in the subgroups, although no obvious difference in the other non-naïve subgroups was observed (Fig. 2B).

**Table 2:** Changes from baseline to week 24 in NT-proBNP concentration

NT-proBNP, pg/mL	Canagliflozin	Glimepiride
All patients	(n = 101)	(n = 109)
Baseline	230.6 (178.2 to 298.3)	205.3 (160.2 to 263.0)
Week 24	225.2 (174.1 to 291.3)	219.5 (171.3 to 281.2)
Proportional change from baseline to week 24	0.98 (0.89 to 1.08)	1.07 (0.97 to 1.18)
Naïve	(n = 36)	(n = 42)
Baseline	302.4 (206.7 to 442.5)	309.1 (217.3 to 439.6)
Week 24	277.3 (189.5 to 405.7)	298.9 (210.1 to 425.2)
Proportional change from baseline to week 24	0.92 (0.78 to 1.08)	0.97 (0.83 to 1.12)
Non-naïve	(n = 65)	(n = 67)
Baseline	198.4 (141.9 to 277.4)	158.8 (114.2 to 220.9)
Week 24	200.6 (143.5 to 280.5)	180.9 (130.1 to 251.6)
Proportional change from baseline to week 24	1.01 (0.89 to 1.14)	1.14 (1.01 to 1.29)
Non-naïve metformin user	(n = 15)	(n = 24)
Baseline	148.6 (67.2 to 328.6)	124.9 (66.7 to 233.9)
Week 24	167.3 (75.7 to 370.1)	158.3 (84.5 to 296.4)
Proportional change from baseline to week 24	1.13 (0.89 to 1.42)	1.27 (1.05 to 1.53)
Non-naïve DPP-4 inhibitor user	(n = 57)	(n = 57)
Baseline	213.9 (149.3 to 306.4)	164.0 (114.5 to 235.0)
Week 24	207.7 (145.0 to 297.5)	181.9 (127.0 to 260.5)
Proportional change from baseline to week 24	0.97 (0.85 to 1.11)	1.11 (0.97 to 1.26)

Data are expressed as the geometric means of NT-proBNP concentration (95% CI) or change in ratio (95% CI)

*CI* confidence interval, *DPP-4* dipeptidyl peptidase-4, *NT-proBNP* N-terminal pro-brain natriuretic peptide

### Effects on SBP, BMI, and ePV

**Fig. 3:**
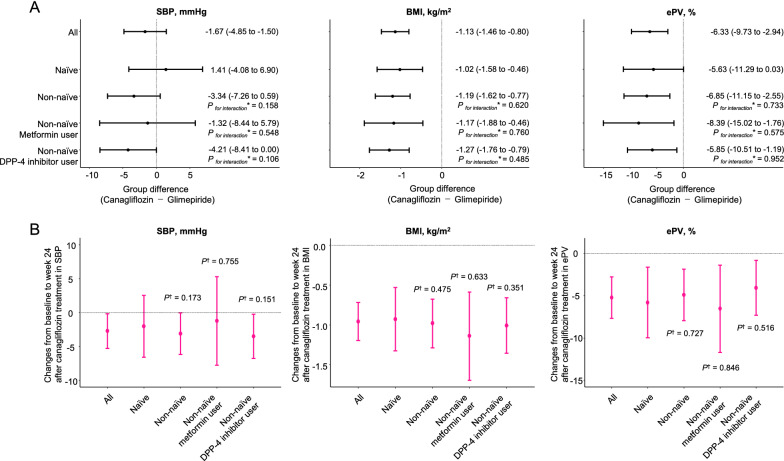
Changes in SBP, BMI, and ePV from baseline to week 24 in the subgroups stratified by the baseline use of glucose-lowering agents. **A** The group differences (canagliflozin—glimepiride) of changes from baseline to week 24 (* refers to the naïve subgroup). The data are expressed as mean (95% confidence interval). **B** Absolute changes from baseline to week 24 after canagliflozin treatment (^†^ refers to the naïve subgroup). *BMI* body mass index, *DPP-4* dipeptidyl peptidase-4, *ePV* estimated plasma volume, *SBP* systolic blood pressure

Changes from baseline to week 24 in SBP, BMI, and ePV, according to the use of glucose-lowering agents at baseline are shown in Table 3 and Fig. 3. No heterogeneity in the treatment effect of canagliflozin, relative to glimepiride, on these parameters was observed in each subgroup, compared to that in the naïve subgroup (Fig. 3A). No significant difference in the impact of canagliflozin on these parameters was observed between the naïve and non-naïve subgroups (Fig. 3B).

**Table 3:** Changes from baseline to week 24 in SBP, BMI, and ePV

Variables	Canagliflozin	Glimepiride
SBP, mmHg		
All patients	(n = 107)	(n = 113)
Baseline	125.1 (122.1 to 128.2)	124.7 (121.8 to 127.7)
Week 24	122.4 (119.3 to 125.4)	123.8 (120.8 to 126.8)
Absolute change from baseline to week 24	− 2.74 (− 5.28 to − 0.20)	− 0.92 (− 3.39 to 1.55)
Naïve	(n = 38)	(n = 43)
Baseline	126.2 (121.4 to 131.0)	123.8 (119.3 to 128.4)
Week 24	124.1 (119.3 to 128.9)	121.5 (116.9 to 126.0)
Absolute change from baseline to week 24	− 2.03 (− 6.59 to 2.54)	− 2.37 (− 6.67 to 1.92)
Non-naïve	(n = 69)	(n = 70)
Baseline	124.5 (120.6 to 128.5)	125.3 (121.4 to 129.2)
Week 24	121.4 (117.5 to 125.3)	125.2 (121.3 to 129.1)
Absolute change from baseline to week 24	− 3.13 (− 6.20 to − 0.06)	− 0.03 (− 3.07 to 3.02)
Non-naïve metformin user	(n = 18)	(n = 26)
Baseline	121.2 (113.6 to 128.9)	122.6 (116.2 to 129.0)
Week 24	120.0 (112.3 to 127.7)	122.1 (115.7 to 128.5)
Absolute change from baseline to week 24	− 1.22 (− 7.74 to 5.29)	− 0.50 (− 5.92 to 4.92)
Non-naïve DPP-4 inhibitor user	(n = 60)	(n = 59)
Baseline	124.5 (120.2 to 128.8)	125.0 (120.7 to 129.3)
Week 24	121.0 (116.7 to 125.3)	125.5 (121.2 to 129.8)
Absolute change from baseline to week 24	− 3.52 (− 6.78 to − 0.25)	0.53 (− 2.77 to 3.82)
BMI, kg/m^2^		
All patients	(n = 109)	(n = 109)
Baseline	25.3 (24.6 to 26.1)	25.7 (25.0 to 26.4)
Week 24	24.4 (23.6 to 25.1)	25.8 (25.1 to 26.6)
Absolute change from baseline to week 24	− 0.96 (− 1.20 to − 0.72)	0.14 (− 0.09 to 0.38)
Naïve	(n = 39)	(n = 42)
Baseline	24.4 (23.3 to 25.5)	25.3 (24.3 to 26.3)
Week 24	23.5 (22.4 to 24.6)	25.4 (24.3 to 26.4)
Absolute change from baseline to week 24	− 0.93 (− 1.33 to − 0.53)	0.05 (− 0.33 to 0.44)
Non-naïve	(n = 70)	(n = 67)
Baseline	25.8 (24.9 to 26.8)	25.9 (24.9 to 26.9)
Week 24	24.9 (23.9 to 25.8)	26.1 (25.1 to 27.1)
Absolute change from baseline to week 24	− 0.98 (− 1.29 to − 0.68)	0.20 (− 0.11 to 0.51)
Non-naïve metformin user	(n = 18)	(n = 25)
Baseline	27.6 (25.6 to 29.6)	26.8 (25.1 to 28.5)
Week 24	26.5 (24.5 to 28.5)	26.9 (25.2 to 28.6)
Absolute change from baseline to week 24	− 1.14 (− 1.70 to − 0.59)	0.09 (− 0.38 to 0.56)
Non-naïve DPP-4 inhibitor user	(n = 61)	(n = 56)
Baseline	25.7 (24.6 to 26.7)	26.3 (25.2 to 27.4)
Week 24	24.7 (23.6 to 25.7)	26.5 (25.4 to 27.6)
Absolute change from baseline to week 24	− 1.01 (− 1.36 to − 0.66)	0.21 (− 0.15 to 0.57)
ePV		
All patients	(n = 107)	(n = 114)
Percent change from baseline to week 24, %	− 5.22 (− 7.66 to − 2.78)	1.11 (− 1.25 to 3.47)
Naïve	(n = 38)	(n = 45)
Percent change from baseline to week 24, %	− 5.80 (− 9.97 to − 1.63)	− 0.17 (− 4.00 to 3.66)
Non-naïve	(n = 69)	(n = 69)
Percent change from baseline to week 24, %	− 4.90 (− 7.94 to − 1.86)	1.95 (− 1.09 to 4.99)
Non-naïve metformin user	(n = 17)	(n = 26)
Percent change from baseline to week 24, %	− 6.53 (− 11.69 to − 1.38)	1.86 (− 2.31 to 6.03)
Non-naïve DPP-4 inhibitor user	(n = 61)	(n = 58)
Percent change from baseline to week 24, %	− 4.08 (− 7.33 to − 0.83)	1.77 (− 1.57 to 5.11)

Data are expressed as means (95% CI)

*BMI* body mass index, *CI* confidence interval, *DPP-4* dipeptidyl peptidase-4, *ePV* estimated plasma volume, *SBP* systolic blood pressure

### Effect on NYHA class

**Fig. 4:**
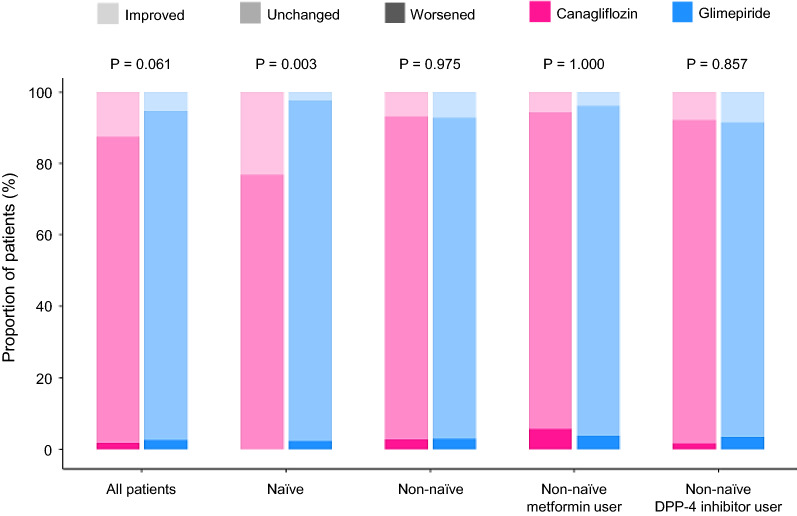
Changes from baseline in NYHA classification at week 24 in the subgroups stratified by the baseline use of glucose-lowering agents. All *p* values were for the comparisons between the treatment groups. *DPP-4* dipeptidyl peptidase-4, *NYHA* New York Heart Association

Categorical changes in NYHA class at week 24, according to the use of glucose-lowering agents at baseline, are shown in Fig. 4. Among the subgroups, a significant difference in the changes in NYHA class between the treatment groups was only observed in the naïve subgroup (canagliflozin vs. glimepiride, *p* = 0.003), while there was no significant difference in NYHA class in the non-naïve subgroup (*p* value of 0.027 for the interaction in treatment effect between naïve vs. non-naïve, calculated in an ordinal logistic regression model.

## Discussion

To the best of our knowledge, this is the first study in patients with T2D and established CHF (almost all with NYHA class I to II and HFpEF) to assess whether the effects of a SGLT2 inhibitor on HF-related clinical parameters differ according to the baseline use of medications for T2D. The major finding of the study was that the effects of canagliflozin treatment on NT-proBNP concentration and relevant markers, except for NYHA class, were unaffected by the baseline use of glucose-lowering agents. This suggests that the clinical effects expected from initiation of a SGLT2 inhibitor on HF-related parameters are independent of the background situation of diabetes therapy, at least in our study patient population.

HF is an important and common complication in patients with T2D, with the coexistence of these two conditions associated with an increased risk of HHF and mortality [21, 22]. There is also evidence that higher levels of HbA1c are associated with an increased risk of incident HF, cardiovascular death, and mortality [23–25]. Conventional diabetes care focuses on optimizing and/or intensifying glycemic control and is not necessarily associated with a reduction in the risk of HF-related events [26]. In contrast, some glucose-lowering agents have been reported to increase the risk of HF [27–29]. Appropriate selection of glucose-lowering agents is crucial for managing the risk of HF in patients with T2D and it is therefore very important to establish treatment strategies that reduce the risk of HF in diabetes care [30].

The findings of a series of CVOTs on SGLT2 inhibitors have led to a reduction in the risk of HF-related events in a broad range of patient populations with cardiovascular and renal risks, irrespective of the patient’s diabetes status [12, 30–32]. Based on these results, the newly-updated treatment guidelines of the ADA recommend considering the use of SGLT2 inhibitors with proven benefits, especially in patients with T2D and concomitant HF, independent of glycemic control and background metformin therapy [13]. This implies that SGLT2 inhibitors may be used more frequently as first-line drugs in specific at risk patient populations. However, to date there is only limited clinical evidence on SGLT2 inhibitor monotherapy, relative to other glucose-lowering agents, in patients with T2D (drug-naïve), irrespective of their CHF history. Furthermore, in accordance with the conventional treatment approach for T2D, many patients with T2D at risk of developing HF, or even those with existing HF, had been taking metformin and/or combination therapy of glucose-lowering agents prior to the initiation of SGLT2 inhibitors. It is therefore clinically important to assess the effect of add-on SGLT2 inhibitor therapy on HF-related clinical parameters according to the background use of glucose-lowering agents. This approach may possibly allow the pragmatic efficacy and safety of that therapy to be predicted in patients treated for both T2D and CHF.

Several CVOTs have confirmed that treatment with SGLT2 inhibitors consistently reduces the risk of HF-related events in patients who had previously been taking glucose-lowering agents, including metformin [14–16]. However, only a small number of studies have evaluated the effects of SGLT2 inhibitors on HF-related clinical parameters, such as NT-proBNP concentration, according to the background use of glucose-lowering medications in patients complicated with HF. The main finding of the present study was that canagliflozin treatment had no different effect on NT-proBNP concentration according to the baseline status of glucose-lowering agents in clinically stable patients with T2D and CHF (almost all with NYHA class I to II and HFpEF).

Importantly, at an individual-trial level some randomized clinical trials, including the CANDLE trial, showed that short-term treatment with SGLT2 inhibitors had a neutral effect on natriuretic peptide concentrations even in patients with established HF [33]. However, a recent meta-analysis demonstrated that treatment with a SGLT2 inhibitor was associated with significant improvements in plasma NT-proBNP concentrations in patients with T2D, irrespective of the presence of CHF [33]. This suggests that the therapeutic impact of SGLT2 inhibitors on HF is not necessarily reflected by changes in NT-proBNP concentration, although the precise reasons for this remain unclear. Interestingly, a recent mediation analysis in the Canagliflozin Cardiovascular Assessment Study (CANVAS) program reported that the decrease in NT-proBNP concentration caused by treatment with a SGLT2 inhibitor was associated with a relatively small reduction in the risk of HF-related events [34]. Therefore, there is an urgent need to establish reliable markers for predicting or monitoring the cardiovascular benefits of SGLT2 inhibitors [35]. In this regard, changes in some fluid volume parameters indicative of a hemodynamic effect of SGLT2 inhibition are likely to be associated with clinical benefits [34, 36]. Recent studies have reported a consistent reduction in ePV status in patients with HFrEF, regardless of their diabetes status, [20] and also in patients with T2D and cardiovascular diseases, regardless of their background clinical characteristics and medications [37]. The present study showed that the effect of canagliflozin treatment on ePV reduction was also consistent across the baseline use of glucose-lowering agents. These findings suggest that measurement of ePV is clinically useful for monitoring the effect of SGLT2 inhibitors on fluid volume status in various situations of diabetes and cardiovascular care. On the other hand, we found that canagliflozin treatment, relative to glimepiride, improved the NYHA class only in the naïve subgroup. Although the explanation for this finding remains uncertain, it is possible that the initial introduction of SGLT2 inhibitor was associated with an improvement in HF-specific symptoms in drug-naïve T2D patients with HF.

The DAPA-HF (Dapagliflozin And Prevention of Adverse Outcomes in Heart Failure) trial showed that treatment with a SGLT2 inhibitor was also effective for reducing the risk of HF-related events and decreasing NT-proBNP concentrations in patients with HFrEF, regardless of whether they had T2D [38]. Of the 2,139 participants with concomitant T2D in that trial, 1596 (74.6%) had been taking background glucose-lowering agents, with 1020 (47.7%) treated at least with metformin [38]. The beneficial impact of dapagliflozin on composite clinical events (worsening HF or cardiovascular death) was consistent across these background medications for T2D [39]. The present study showed that the effects of canagliflozin treatment on the HF-related parameters examined were also unaffected by baseline use of metformin treatment, although it is important to note that the baseline prescription rate of metformin was low in our study. Metformin is recognized as being clinically safe at all stages of HF with preserved or stable, moderately reduced renal function [11], and is associated with a lower incidence of HF-related events and mortality in patients with T2D and HF [6, 7, 40]. Nevertheless, metformin is still contraindicated in patients with severe cardiac dysfunction and HF in Japan. This may partially explain the lower rate of baseline metformin use and higher rate of baseline DPP-4 inhibitor use in the clinically stable cohort of the CANDLE trial. Given their safety and efficacy for glycemic control, DPP-4 inhibitors are still prescribed frequently in Asian populations with T2D, even in patients with HF [41–43]. The present study showed that the baseline use of DPP-4 inhibitors did not influence the effects of canagliflozin treatment on the clinical variables examined. These results partially support the non-glycemic safety and clinical efficacy of combination therapy with DPP-4 inhibitors and SGLT2 inhibitors [44–46].

Several limitations need to be considered when interpreting our findings. First, the study was a post hoc analysis of the CANDLE trial that was not designed specifically or powered to perform subgroup analysis according to baseline therapy. The trial was also an open-label design and therefore the study outcomes might have been affected, in part, by the local investigators’ clinical performance. Second, we cannot exclude the possibility that insufficient variability in NT-proBNP concentrations due to the use of a natural logarithmic scale may have potentially influenced our findings. Although this method for analyzing NT-proBNP concentrations has been used in several other clinical trials on HF that assessed the interventional impact on NT-proBNP concentration as a surrogate endpoint of HF treatment [47, 48], it is still controversial whether measuring natriuretic peptides is sufficient to identify a clinically meaningful treatment effect [49, 50]. Moreover, the magnitude of change in NT-proBNP concentration that is considered to be of clinical significance remains uncertain. Due to the exploratory nature of this post hoc substudy of the CANDLE trial we did not pre-define a specific level of change in NT-proBNP concentration that would be classified as clinically significant. Third, the dose of canagliflozin was limited to 100 mg daily due to regulations in Japan, while the dose of glimepiride at the final visit was low (median 1.0 mg daily, interquartile range 0.5–1.0). Fourth, there was no patient-level information provided on the clinical context of the study that may have influenced drug use at the time of enrollment. We are therefore unable to exclude the possibility that some background differences between the cohorts and unknown confounding factors might have influenced the outcome measures. In addition, the current findings were also not adjusted for changes in non-study medications during the trial, including other glucose-lowering agents and therapies for HF. Finally, the CANDLE trial only included Japanese patients with clinically stable T2D and CHF (almost all with NYHA class I to II and HFpEF), and it is therefore uncertain whether these findings are applicable to other ethnicities and different clinical severities and phenotypes of HF.

## Conclusion

Our findings suggest that the impact of canagliflozin treatment on NT-proBNP concentration and its beneficial effect on volume-related parameters appear to be independent of the background use of diabetes therapy in the patient population in this study. On the other hand, canagliflozin treatment improved NYHA class only in patients who had not been taking glucose-lowering agents. Further studies are warranted to confirm these findings and to assess the detailed clinical impact of SGLT2 inhibitors according to different medical situations.

## Supplementary Information

**Additional file 1.** Baseline demographic and clinical characteristics of each study cohort.

## Data Availability

The datasets analyzed during the current study are available from the corresponding author on reasonable request (tanakaa2@cc.saga-u.ac.jp).

## Abbreviations

ASCVD: Atherosclerotic cardiovascular disease
BMI: Body mass index
CHF: Chronic heart failure
CI: Confidence interval
CVOT: Cardiovascular outcome trial
DPP-4: Dipeptidyl peptidase-4
EASD: European Association for the Study of Diabetes
ESC: European Society of Cardiology
HF: Heart failure
HFpEF: Heart failure with preserved ejection fraction
HHF: Hospitalization for heart failure
NT-proBNP: N-terminal pro-brain natriuretic peptide
NYHA: New York Heart Association
SBP: Systolic blood pressure
SGLT2: Sodium–glucose cotransporter 2
