# Supplementary material for: Effect of canagliflozin on N-terminal pro-brain natriuretic peptide in patients with type 2 diabetes and chronic heart failure according to baseline use of glucose-lowering agents
Source: Cardiovasc Diabetol. 2021 Sep 3;20:175. doi: 10.1186/s12933-021-01369-5 (PMC8417990; doi:10.1186/s12933-021-01369-5)
Supplement: Supplementary file 1 — Additional file 1. Baseline demographic and clinical characteristics of each study cohort. [file 12933_2021_1369_MOESM1_ESM.docx]

**Additional file 1. Baseline demographic and clinical characteristics of each study cohort**

| **Variables** | **Overall**  **(n = 233)** | **Naïve**  **(n = 85)** | **Non-naïve**  **(n = 148)** | **Standardized difference*** |
| --- | --- | --- | --- | --- |
| Age, yr | 68.6 ± 10.1 | 70.2 ± 9.5 | 67.6 ± 10.3 | 0.258 |
| Males | 174 (74.7) | 62 (72.9) | 112 (75.7) | 0.062 |
| Body mass index, kg/m^2^ | 25.5 ± 3.9 | 25.0 ± 3.6 | 25.8 ± 4.1 | 0.189 |
| Systolic blood pressure, mmHg | 124.7 ± 16.3 | 125.1 ± 15.2 | 124.5 ± 16.8 | 0.037 |
| History |  |  |  |  |
| Hypertension | 102 (43.8) | 37 (43.5) | 65 (43.9) | 0.008 |
| Dyslipidemia | 100 (42.9) | 40 (47.1) | 60 (40.5) | 0.131 |
| Myocardial infarction | 56 (24.0) | 19 (22.4) | 37 (25.0) | 0.062 |
| Angina pectoris | 51 (21.9) | 19 (22.4) | 32 (21.6) | 0.018 |
| Heart failure cause |  |  |  |  |
| Ischemia | 100 (42.9) | 33 (38.8) | 67 (45.3) | 0.130 |
| Heart failure status |  |  |  |  |
| NYHA class |  |  |  |  |
| I | 148 (63.5) | 48 (56.5) | 100 (67.6) | 0.229 |
| II | 79 (33.9) | 34 (40.0) | 45 (30.4) | 0.201 |
| III | 5 (2.1) | 2 (2.4) | 3 (2.0) | 0.022 |
| Unknown | 1 (0.4) | 1 (1.2) | 0 (0.0) | 0.153 |
| LVEF distribution |  |  |  |  |
| <30% | 13 (5.6) | 4 (4.7) | 9 (6.1) | 0.062 |
| 30 to <40% | 20 (8.6) | 8 (9.4) | 12 (8.2) | 0.044 |
| 40 to <50% | 34 (14.7) | 11 (12.9) | 23 (15.6) | 0.077 |
| ≥50％ | 165 (71.1) | 62 (72.9) | 103 (70.1) | 0.063 |
| Medications |  |  |  |  |
| Non-diabetic |  |  |  |  |
| ACE inhibitor or ARB | 177 (76.0) | 58 (68.2) | 119 (80.4) | 0.280 |
| Beta-blocker | 164 (70.4) | 67 (78.8) | 97 (65.5) | 0.298 |
| MRA | 86 (36.9) | 34 (40.0) | 52 (35.1) | 0.100 |
| Diuretic | 99 (42.5) | 40 (47.1) | 59 (39.9) | 0.145 |
| Diabetic |  |  |  |  |
| Insulin | 7 (3.0) | 0 (0.0) | 7 (4.7) | - |
| Metformin | 44 (18.9) | 0 (0.0) | 44 (29.7) | - |
| DPP-4 inhibitor | 127 (54.5) | 0 (0.0) | 127 (85.8) | - |
| Others | 41 (17.6) | 0 (0.0) | 41 (27.7) | - |

Data are expressed as the mean ± standard deviation or n (%).

* Naïve vs. non-naïve.

ACE, angiotensin-converting enzyme; ARB, angiotensin receptor blocker; DPP-4, dipeptidyl peptidase-4; LVEF, left ventricular ejection fraction; MRA, mineralocorticoid receptor antagonist; NYHA, New York Heart Association.
